# A Randomized Controlled Trial on the Efficacy of Xinnaoning Capsule in the Treatment of CSAP Complicated With Qi Stagnation and Blood Stasis Syndrome

**DOI:** 10.3389/fcvm.2022.859956

**Published:** 2022-06-16

**Authors:** Jing Li, Ping Zhang, Ying Zhang, Haiyun Wang, Liqi Wu, Junnan Zhao, Yue Liu, Wenying Zeng, Renzhen Guo, Jun Mei, Fengqin Xu

**Affiliations:** ^1^Department of General Medicine, Cardiovascular Direction of Integrated Traditional Chinese and Western Medicine, Xiyuan Hospital, Chinese Academy of Traditional Chinese Medicine (TCM), Beijing, China; ^2^Department of Cardiovascular, Cardiovascular Direction of Integrated Traditional Chinese and Western Medicine, Xiyuan Hospital, Chinese Academy of Traditional Chinese Medicine (TCM), Beijing, China

**Keywords:** Xinnaoning Capsule, chronic stable angina pectoris, RCT, Qi stagnation, blood stasis syndrome

## Abstract

**Objective:**

This study was designed to explore the efficacy and safety of Xinnaoning Capsule (XNNC) in the treatment of patients with chronic stable angina pectoris (CSAP) complicated with Qi stagnation and blood stasis syndrome.

**Methods:**

A total of 240 patients with CSAP complicated with Qi stagnation and blood stasis syndrome who met the inclusion criteria were enrolled from 8 medical centers across China. The trial treatment lasted 14 weeks, including a 2-week lead-in period and a 12-week double-blind treatment period. Patients in the experimental group were treated with XNNC, while patients in the control group were treated with placebos. Thereafter, examinations were conducted on the efficacy of angina pectoris before and after treatment and the relief of symptoms, followed by the recording of grading changes in angina severity, changes in the number of angina pectoris, and the amount of taken nitroglycerin. Finally, adverse events were assessed.

**Results:**

Compared with the control group, the total score and the effective rate of angina pectoris were significantly increased in the experimental group, accompanied by the statistically significant improvement in the severity of angina pectoris (all *p* < 0.05). Furthermore, there was no statistically significant difference in the adverse events and serious adverse events between the experimental group and the control group (*p* = 1.0000) before and after treatment.

**Conclusion:**

XNNC is a safe and effective medicine for patients with CSAP complicated with Qi stagnation and blood stasis syndrome.

## Introduction

Cardiovascular disease is one of the leading causes of death in China. Moreover, it is estimated that 23 million people will suffer from acute myocardial infarction by 2030 in China (1). Traditional Chinese Medicine (TCM) treatment has unique advantages in alleviating the symptoms and improving the prognosis of angina pectoris. TCM emphasizes the principles of “treat both the symptoms and the root causes”, “prevent the disease”, and “prevention and recovery”. In addition, it protects blood vessels, improves microcirculation, and prevents platelet aggregation. Therefore, TCM exerts various effects, such as anti-inflammation, anti-atherosclerosis, anti-oxidation, and oxygen consumption reduction. The integration of traditional Chinese and western medicine treatments can better alleviate the clinical symptoms of patients and reduce restenosis and angina pectoris after stent surgery, as well as decrease the incidence of adverse events (2, 3). In addition, it has been reported that the long-term use of proprietary Chinese medicines can improve the prognosis of patients undergoing coronary artery bypass grafting or interventional therapy (4, 5).

In the formula of Xinnaoning Capsule (XNNC), Ginkgo biloba is utilized as the monarch medicine to promote blood circulation, remove blood stasis, and relieve pain. This formula is applied for patients with coronary heart disease (CHD), angina pectoris, and hyperlipidemia. Furthermore, previous studies have revealed that XNNC can obviously relieve chest pain, chest tightness, fatigue, and other symptoms in patients with chest or angina pectoris complicated with Qi stagnation and blood stasis syndrome after coronary stent implantation. More importantly, XNNC shows certain advantages of improving myocardial microcirculation, facilitating the opening of collateral circulation, inhibiting platelet aggregation, lowering blood lipids, and stabilizing the plate for patients with angina pectoris after coronary stent implantation (6–14).

Accordingly, we intend to design a randomized, double-blind, parallel-controlled, and multi-center clinical trial to evaluate the effectiveness and safety of XNNC in the treatment of patients with chronic stable angina pectoris (CSAP) complicated with Qi stagnation and blood stasis syndrome (placebo acted as the control). The efficacy of XNNC on angina pectoris was observed by objectively evaluating the number of attacks, the incidence of cardiovascular events, and other indicators, such as the effectiveness, safety, and long-term benefits of XNNC.

## Materials and Methods

### CHD Diagnostic Criteria

#### Western Medicine Diagnostic Criteria

Patients meeting the following criteria were classified as CHD according to 2012 ACP/ACCF/AHA/AATS/PCNA/STS Diagnosis of Stable Ischemic Heart Disease: Clinical Practice Guidelines released by the European Society of Cardiology (ESC) in 2013 and the setting of “Guidelines for the Management of Stable Coronary Artery Disease” combined with the “Guidelines for the Diagnosis and Treatment of Chronic Stable Angina Pectoris” launched by the Chinese Medical Association in 2007 (15).

(1) Patients had a definite history of myocardial infarction or a history of percutaneous coronary intervention (PCI) or bypass;
(2) Coronary angiography results showed stenosis on at least 1 side of the coronary artery or more than half of the stenosis area, or the coronary computed tomography angiography (CTA) examination displayed the degree of lumen stenosis of not <50%.

#### TCM Diagnostic Criteria

Based on “Guiding Principles for Clinical Research of Chinese Medicines and Natural Medicines for CHD and Angina Pectoris” in 2011 and “Guiding Principles for Clinical Research of New Chinese Medicines” (2002 Edition), TCM syndrome differentiation of chest obstruction were conducted with the following criteria: (1) chest pains, even chest pains all over the back; (2) mild symptoms, like chest tightness, suffocation, and shortness of breath.

In addition, Qi stagnation and blood stasis syndrome had the following symptoms:

(1) Main symptoms: chest pain and chest tightness;
(2) Secondary symptoms: fullness in the chest and flanks, palpitations, shortness of breath, dark purple lips (the darker the lip, the severer the blood stasis), fatigue, dizziness, dark purple veins under the tongue, and irritability;
(3) Tongue image: dark tongue (the darker the tongue, the severe blood stasis);
(4) Pulse condition: astringent (a sign of blood stasis).

Patients with one of the main symptoms or two of the secondary symptoms and tongue and pulse support were diagnosed. TCM syndrome scoring method is shown in Table 1.

**Table 1:** TCM syndrome scoring.

		**Score**
Main symptom	Chest pain	0: No 2: relieved by rest, does not affect daily life 4: drug required during the attack, and normal activities can be continued after remission 6: frequent attacks, affecting activities in daily life (such as dressing, eating, walking, defecation)
	Chest tightness	0: No 2: Occasional, relieved without interfering 4: frequently, does not affect life and work 6: continuesly, affect normal life and work
Secondary symptoms	Palpitations	0: No 1: Occasional, relieved without interfering 2: frequently, able to keep working 3: continuesly, affect normal life and work
	Shortness of breath	0: No 1: after general activities 2: after slight activity 3: breath shortness without activities
	Fullness of the chest and flanks	0: No 1: occasionally, relieved without interfering 2: long fullness time, medicine required 3: recurrent attacks, medicine required to alleviate
	Fatigue	0: No 1: Low spirit, poor strength, can adhere to the daily work and activities 2: Weak spirit and strength, manage with an effort to work 3: severe lack of spirit and strength, barely work
	Dark purple lips	0: No 1: Yes
	Dizziness	0: No 1: Yes
	Dark purple veins under the tongue	0: Light purple 1: dark purple, angiectasis, extravasated blood point
	Irritability	0: No 1: Yes
Tongue image		Dark tongue Other _____
Pulse condition		Astringent Other _____

*Tongue image and pulse condition is only described and not scored*.

### Inclusion Criteria

(1) According to the “Guidelines for the Diagnosis and Treatment of Chronic Stable Angina Pectoris”, 2012 ACP/ACCF/AHA/AATS/PCNA/STS Diagnosis of Stable Ischemic Heart Disease: Clinical Practice Guidelines released by ESC in 2013, and Guidelines for Stable Coronary Artery Disease Management, the guidelines were formulated to meet the following criteria in order to diagnose the disease:
  (a) There must be an old history of myocardial infarction or a history of PCI or bypass;
  (b) Coronary angiography results clearly indicated that there was coronary artery stenosis with the stenosis range > 50% or coronary CTA exhibited that the stenosis range was >50%;
(2) CSAP patients met the criteria of the diagnosis: an onset history of more than 30 days and no significant change in the degree, frequency, nature, and predisposing factors of the disease;
(3) Patients with the severity of angina pectoris from grade I to grade III classified by the Canadian Cardiovascular Society (CCS) and the number of attacks per week of ≥2;
(4) Patients with Qi stagnation and blood stasis syndrome based on TCM differentiation;
(5) Patients aged between 30 and 79 years;
(6) Patients signing the informed consent form.

### Exclusion Criteria

(1) Patients with severe cardiopulmonary insufficiency (grade III and IV of heart function abnormality or severe lung function abnormality);
(2) Patients with unstable blood pressure control (systolic blood pressure ≥160 mmHg or diastolic blood pressure ≥100 mmHg after diagnosis and treatment);
(3) Patients with a combination of severe alanine aminotransferase and aspartate aminotransferase ≥1.5 times the upper limit of normal or Cr > upper limit of normal, accompanied by severe hematopoietic system diseases;
(4) Patients with the interventional diagnosis and treatment of acute myocardial infarction within 3 months;
(5) Patients with other reasons that led to changes in the electrocardiogram ST-T, such as tetralogy of Fallot;
(6) Patients with a pacemaker;
(7) Patients with chest pain caused by other reasons (moderate or above anemia or hyperthyroidism);
(8) Women who were pregnant or breastfeeding or planning to become pregnant;
(9) Patients with allergies or allergies to the known ingredients of the research drug;
(10) Participants in other clinical drug trials within the past month;
(11) Patients considered by the investigator to be unsuitable for participation in this trial.

### Patients

This study enrolled 240 outpatients with CSAP complicated with Qi stagnation and blood stasis syndrome who met the inclusion criteria from June 2018 to December 2019. Among them, there were 120 cases in the control group and 120 cases in the experimental group. The research subjects were from the Principal Study Site, China Academy of Chinese Medical Sciences Xiyuan Hospital, and other centers (Xi'an TCM Hospital, Xidian Group Hospital, Shanghai University of TCM Affiliated Shuguang, Second Affiliated Hospital of the Shandong University of TCM, Shaanxi Provincial Hospital of TCM, First Affiliated Hospital of the Henan University of TCM, and Second Affiliated Hospital of Luoyang City). This experimental study passed the Good Clinical Practice (GCP) ethics review (the ethical approval number: 2017XL027-2; the clinical trial protocol registration number: NCT 03914131) (16).

The trial period consisted of a lead-in period of 2 weeks and a double-blind treatment period of 12 weeks. XNNC (batch number: 20170916) and placebo (batch number: 20171113) were provided by Guizhou Jingcheng Pharmaceutical Company (0.45 g/capsule, Guizhou, China). During the treatment period, patients in the experimental group were administered 3 tablets of XNNC, thrice a day, while patients in the control group took 3 tablets of placebos, thrice a day. During the lead-in period and the treatment period, patients in the experimental group and the control group could use antiplatelet drugs, angiotensin-converting enzyme inhibitors or angiotensin II receptor blockers, statins, lipid-lowering drugs, calcium antagonists, and or beta receptors. In addition, body blockers and long-acting nitrate esters could be administered with the unchange of the original dose. Also, Nitroglycerin (a dose of 0.5 mg) could be given sublingually when angina pectoris occurred but could be tolerated. The number and dosage of medications were recorded in detail.

### Observation Indexes

#### Main Efficacy Indicators: The Efficacy of Angina Pectoris Symptoms

According to the angina pectoris symptom scoring method, patients in the 2 groups were compared before and after angina pectoris symptom scoring. The angina pectoris symptom scoring method is presented in Table 2:

(1) Significantly effective: *n* ≥ 70%;
(2) Effective: 30% ≤ *n* < 70%;
(3) Invalid: 0 ≤ *n* < 30%;
(4) Increased: *n* < 0.

**Table 2:** Angina pectoris symptom scoring.

**Symptom**	**Level**	**Score**
1. Frequency of attacks:	None	0
	Every week	2
	1–3 times/day. Or labor class II	4
	>4 times/day. Or labor class III	6
2. Duration of pain:	None	0
	≤ 5 min	1
	>5 and <10 min	2
	≥10 min	3
3. Pain degree:	None	0
	Not severe, no impact on daily life.	1
	Heavier, nitroglycerin is required.	2
	Severe attacks, affect daily living activities (such as dressing, defecating, etc.)	3
4. Dose of nitroglycerin	None	0
	1–4 tablets/week	1
	5–9 tablets/week	2
	>10/week	3

#### Secondary Efficacy Indexes

(1) Changes in the severity grading of angina pectoris;
(2) Changes in the number of angina pectoris attacks per week;
(3) Nitroglycerin dosage;
(4) The incidence of cardiovascular events.

#### Safety Indexes

Safety indexes were examined, including blood routine, urine routine, stool routine, liver and kidney function, blood lipids, blood coagulation four items, and adverse events.

### Sample Size

The sample size was estimated as per statistical requirements, and the efficacy of angina pectoris symptoms after 12-week treatment was the major effect indicator. In the light of the literature, the total effective rate of basic treatment placebo was 67.5%, whereas the total effective rate of basic treatment test drug was expected to be 84.3%. With α = 0.05 and 1-β = 0.2, a group was set up as per the ratio of the experimental group to the control group of 1:1:


(1) $$\begin{array}{l} n=\frac{P_{1}\times \left(100-P_{1}\right)+P_{2}\times \left(100-P_{2}\right)}{{\left({P_{2-}P}_{1}\right)}^{2}}\times f\left(a,b\right) \end{array}$$

The minimum sample size that met the statistical requirements was 99 cases in each group. Considering the dropout rate of no more than 20%, there were 120 cases in each group, for a total of 240 cases.

### Randomization and Blinding

The subjects were randomly assigned into two groups using a block randomization method. The information of 240 subjects (experimental drugs and control drugs) was randomized using the SAS 9.4 statistical software. Following the principle of randomization, each center was assigned a drug number. During the trial, the researchers and subjects were strictly blinded.

### Quality Control

#### Clinical Trial Records

The researcher conformed to the design requirements of the “Case Report Form” and filled all of the items carefully. All laboratory data in clinical trials were recorded and the original report (or copy) was pasted to the records. Information that was significantly higher or outside the clinically acceptable range was verified, and necessary explanations were made by physicians participating in this clinical trial.

#### Management of Trial Drugs

The trial drugs were stored and managed by the trial department, and specific personnel took charge of the storage, distribution, recycling, and returning of drugs. The trial drugs were recycled for withdrawal and loss to follow-up. Meanwhile, the drug usage in clinical trials was recorded, including the distribution date and the number of drugs, the name of subjects, the recycle date and the number of drugs, and the signature of drug providers and drug receivers.

#### Researcher Training

The clinical trial participants were uniformly trained before the start of clinical trials, so that the researchers fully understood the specific connotation and each indicator of the clinical trial scheme, formed the same quantitative standards of symptoms and signs, had the objective description of self-conscious symptoms, checked all of the stated objective indicators using the time and methods of the program, were familiar with the recording methods of medical records and case report form, and were cautious of adverse reactions and follow-up.

#### Measurement of Improving Observation Consistency

(1) Qualification examination for researchers participating in the clinical trials was conducted: they had the professional expertise, qualifications, and ability to conduct clinical trials, and the personnel was relatively fixed after qualification examination.
(2) The researcher verified the data that were obviously biased or outside the acceptable range, and the researcher made necessary explanations.
(3) Each test item was marked as the measurement unit.
(4) Each clinical trial department designated specific personnel to regularly check the progress of clinical trials and carefully confirm data and records.
(5) If necessary, the sponsor organized and held a mid-term clinical meeting to check the preliminary work, analyze the problems during the clinical trial, and make rectification.

#### Supervision of Clinical Trials

The sponsor appoint qualified inspectors to regularly inspect each trial center to ensure that the rights and interests of the subjects were protected, that the data were accurate and complete, and that the trials obeyed the approved protocol, drug clinical trial management norms, and relevant laws and regulations. Inspectors were granted direct access to source documents (raw documents, data, and records), including permission to examine, analyze, and verify crucial records and reports of clinical trials. Researchers kept in touch at all times by telephone for discussions.

### Statistical Analysis

The calculation in the statistical analysis was implemented using the SAS9.4 software. The main efficacy data set was the Full Analysis Set (FAS) analysis population, and the efficacy of the Per Protocol Set (PPS) population was also analyzed.

Mean and standard deviations, as well as maximum, minimum, median, and 95% confidence interval (CI), were employed to describe continuity variables, while frequency or percentage were adopted to describe classification variables. The Student's *t*-test was utilized for the comparison between the two groups, and the chi-square test was applied for the comparison of rates. Cochran-Mantel-Haenszel (CMH) chi-square test regarding central effect was used for the comparison between groups, and 95% *CI* was calculated for the rate difference between the two groups. *p* < 0.05 and the discrimination represented statistically significant differences.

## Results

### General Information

**Figure 1:**
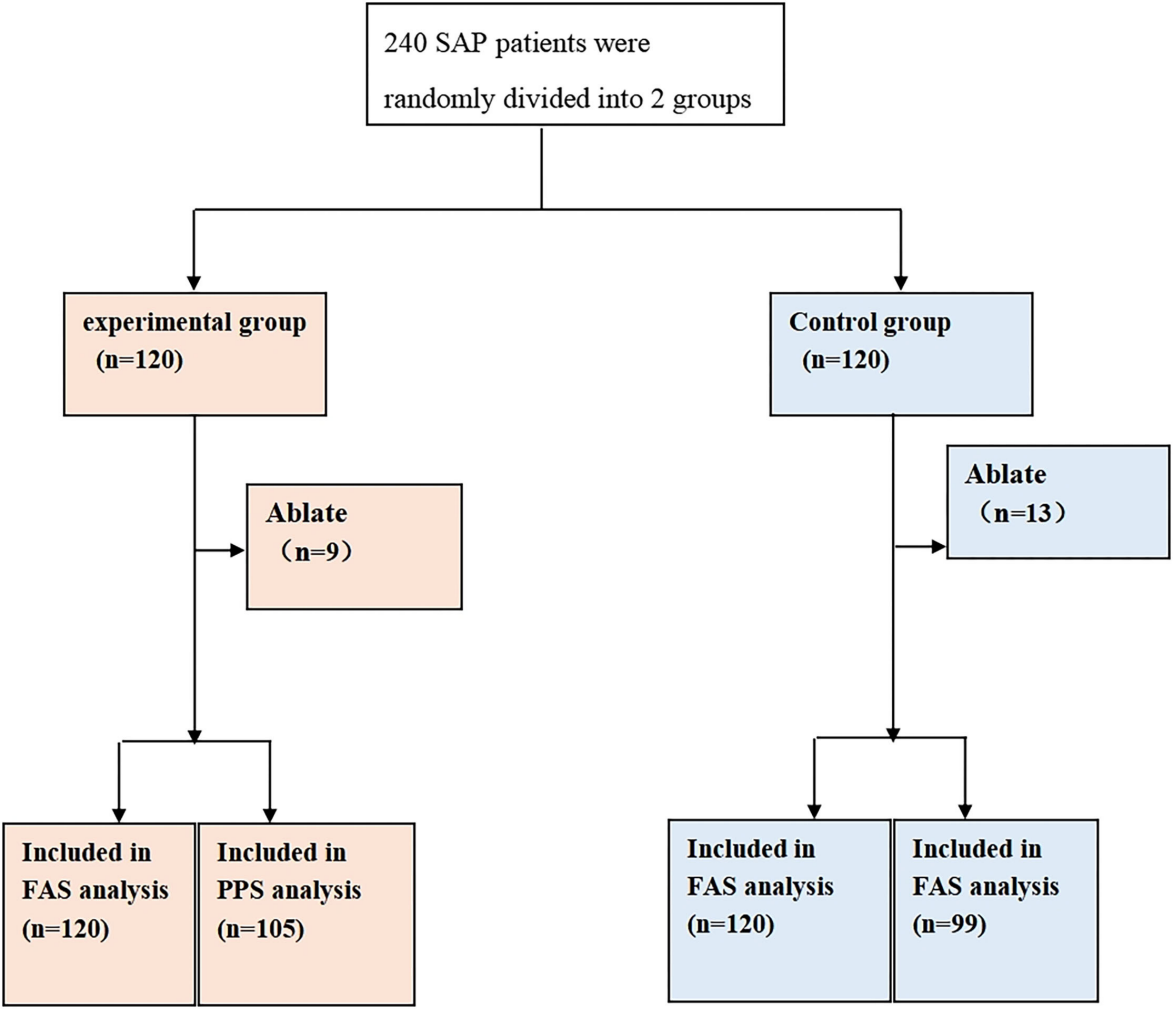
Randomization grouping. A total of 240 patients who met the criteria were recruited and randomly arranged into 2 groups, including 120 patients in the experimental group and 120 in the control group. As for the experimental group, 9 cases were dropout, with 120 patients in the FAS analysis and 105 in the PPS analysis. For the control group, 13 cases were dropout, with 120 patients in the FAS analysis and 99 in the PPS analysis.

The study recruited 240 CSAP patients complicated with Qi stagnation and blood stasis syndrome who met the criteria. Specifically, 120 patients were assigned in the experimental group and 120 patients in the control group. Additionally, there existed 9 dropout cases in the experimental group with a dropout rate of 7.50%, of which 5 cases were lost to follow-up, 2 cases withdrew for other reasons, and 2 cases had adverse events. In the control group, 13 cases dropped out corresponding to a dropout rate of 10.83%, among which 8 cases were lost to follow-up and could not be contacted, 2 cases withdrew with their informed consent withdrawn, 2 cases had adverse events, and 1 case was lack of efficacy. All information is listed in Figure 1. Furthermore, 240 cases were in both FAS and Safety Set populations, with 120 cases in the experimental group and 120 cases in the control group. Moreover, there were 204 cases in the PPS population, including 105 cases in the experimental group and 99 cases in the control group. No statistically significant difference was observed in the dropout rate between the 2 groups (Chi-square = 0.80, *p* = 0.3709, Table 3).

**Table 3:** Comparison of dropout rates between the two groups.

**Project**	**Experimental group**	**Control group**	**Statistics**	***P*-value**
Dropout			*X*^2^ = 0.80	0.3709
Case (missing)	120 (0)	120 (0)		
Finished	111 (92.50%)	107 (89.17%)		
Dropout	9 (7.50%)	13 (10.83%)		

The experimental group had 84 male patients and 36 female patients. The average course of the disease, body mass index (BMI), and body weight were 40.28 ± 47.59 months, 24.53 ± 2.67, and 69.14 ± 9.30 kg, respectively. In the control group, there were 77 men and 43 women, accounting for 64.17 and 35.83%, respectively. The average courses of the disease, BMI, and body weight were 39.45 ± 47.41 months, 24.39 ± 2.45, and 67.82 ± 8.84 kg, respectively. There was no statistically significant difference between the 2 groups in terms of height, weight, BMI, race, gender, and the course of the disease (*p* > 0.05, Table 4).

**Table 4:** Baseline comparison of demographic data between the two groups.

**Project**	**Experimental group**	**Control group**	**Statistics**	***P*-value**
Height			*t* = 1.23	0.2216
Number of cases (missing)	120 (0)	120 (0)		
Mean (standard deviation)	167.76 (6.90)	166.60 (7.72)		
Minimum, maximum	150,182	145, 182		
Median (Q1, Q3)	170 (162, 172)	168 (160, 173)		
The average 95% CI	(166.51, 169.01)	(165.21, 167.99)		
weight			*t* = 1.13	0.2592
Number of cases (missing)	120 (0)	120 (0)		
Mean (standard deviation)	69.14 (9.30)	67.82 (8.84)		
Minimum, maximum	50, 99.50	48, 92.50		
Median (Q1, Q3)	68.65 (62.5, 75)	68 (60.5, 75)		
The average 95% CI	(67.46, 70.82)	(66.22, 69.41)		
**BMI**			*t* = 0.43	0.6685
Number of cases (missing)	120 (0)	120 (0)		
Mean (standard deviation)	24.53 (2.67)	24.39 (2.45)		
Minimum, maximum	18.30, 32.40	18.8, 33.70		
Median (Q1, Q3)	24.25 (22.95, 26.00)	24.2 (23, 25.45)		
The average 95% CI	(24.05, 25.01)	(23.95, 24.83)		
nation			Correction chi-square = 0.82	0.3661
Case (missing)	120 (0)	120 (0)		
Han nation	116 (96.67%)	119 (99.17%)		
Other	4 (3.33%)	1 (0.83%)		
gender			Chi-square = 0.92	0.3363
Case (missing)	120 (0)	120 (0)		
Male	84 (70.00%)	77 (64.17%)		
Female	36 (30.00%)	43 (35.83%)		
Disease period (month)			*t* = 0.13	0.8931
Number of cases (missing)	120 (0)	120 (0)		
Mean (standard deviation)	40.28 (47.59)	39.45 (47.41)		
Minimum, maximum	1, 240	1, 240		
Median (Q1, Q3)	24 (12, 48)	24 (8, 52)		
The average 95% CI	(31.67, 48.88)	(30.88, 48.02)		

### Analysis Results of the Main Effectiveness Indicators

#### Comparison of Total Scores of Angina Pectoris Symptoms

The angina pectoris symptom score was 4.38 ± 2.16 in the experimental group and 4.67 ± 1.77 in the control group in the 4th week (*p* = 0.0267).

The angina pectoris symptom score was 3.33 ± 2.04 in the experimental group and 4.21 ± 1.63 in the control group in the 8th week (*p* = 0.0003), and 2.26 ± 2.50 in the experimental group and 4.08 ± 1.60 in the control group at 12th week after treatment (*p* < 0.0001, Table 5).

**Table 5:** Comparison of total scores of angina pectoris symptoms during the follow-up period (FAS).

**Index**	**Experimental group**	**Control group**	***t***	***P*-value**
Baseline score			*t* = 1.0092	0.3139
Case number (missing)	120 (0)	120 (0)		
Mean (±SD)	5.25 (1.47)	5.06 (1.47)		
Minimum, maximum	4, 9	4, 10		
Median (Q1, Q3)	5 (4, 6)	4 (4, 6)		
Average 95% CI	(4.98, 5.52)	(4.79, 5.32)		
4-week follow-up Scores of Angina Pectoris Symptoms			Corrected *t* = 1.1108	0.2678
Case number (missing)	120 (0)	120 (0)		
Mean (±SD)	4.38 (2.16)	4.67 (1.77)		
Minimum, maximum	0, 14	0, 9		
Median (Q1, Q3)	4 (4, 5)	4 (4, 6)		
Average 95% CI	(3.99, 4.77)	(4.35, 4.99)		
Baseline vs. 4-week data			Corrected *t* = 2.3126	0.0218
Case number (missing)	120 (0)	120 (0)		
Mean (±SD)	0.87 (1.91)	0.39 (1.20)		
Minimum, maximum	−10, 6	−2, 6		
Median (Q1, Q3)	0 (0, 2)	0 (0, 0)		
Average 95% CI	(0.52, 1.21)	(0.18, 0.61)		
Paired *t*-test (*p*)	4.98 (<0.0001)	3.58 (0.0005)		
8-week follow-up			Corrected *t* = 3.7086	0.0003
Case number (missing)	120 (0)	120 (0)		
Mean (±SD)	3.33 (2.04)	4.21 (1.63)		
Minimum, maximum	0, 9	0, 9		
Median (Q1, Q3)	4 (2, 4.5)	4 (4, 5)		
Average 95% CI	(2.96, 3.69)	(3.91, 4.50)		
Baseline vs. 8-week data			Corrected *t* = 2.3126	0.0218
Case number (missing)	120 (0)	120 (0)		
Mean (±SD)	0.87 (1.91)	0.39 (1.20)		
Minimum, maximum	−10, 6	−2, 6		
Median (Q1, Q3)	0 (0, 2)	0 (0, 0)		
Average 95% CI	(0.52, 1.21)	(0.18, 0.61)		
Paired *t*-test (*p*)	4.98 (<0.0001)	3.58 (0.0005)		
12-week follow-up			Corrected *t* = 6.7017	<0.0001
Case number (missing)	120 (0)	120 (0)		
Mean (±SD)	2.26 (2.50)	4.08 (1.60)		
Minimum, maximum	0, 14	0, 9		
Median (Q1, Q3)	2 (0, 4)	4 (4, 5)		
Average 95% CI	(1.81, 2.71)	(3.78, 4.37)		
Baseline vs. 12-week data			Corrected *t* = 7.6238	<0.0001
Case number (missing)	120 (0)	120 (0)		
Mean (±SD)	2.99 (2.43)	0.98 (1.56)		
Minimum, maximum	−10, 8	−2, 5		
Median (Q1, Q3)	4 (1, 4)	0 (0, 2)		
Average 95% CI	(2.55, 3.43)	(0.70, 1.26)		
Paired *t*-test (*p*)	13.48 (<0.0001)	6.92 (<0.0001)		

#### Improvement of Angina Pectoris Symptoms

(1) Curative effect of angina pectoris symptoms

The experimental group showed a markedly effective rate of 49.17% (59 cases), an effective rate of 21.67% (26 cases), an ineffective rate of 27.50% (33 cases), and an aggravated rate of 1.67% (2 cases). However, the control group had a markedly effective rate of 10.00% (12 cases), an effective rate of 15.00% (18 cases), an ineffective rate of 71.67% (86 cases), and an aggravated rate of 3.33% (4 cases). The difference between the 2 groups was statistically significant (CMH-Chisq = 44.08, *p* < 0.0001, Table 6).

**Table 6:** Comparison of curative effect of angina pectoris symptoms.

**Project**	**Experimental group**	**Control group**	**statistics**	***P*-value**
FAS			CMH-Chisq = 44.08	<0.0001
Case (missing)	120 (0)	120 (0)		
Efficacy (%)	59 (49.17%)	12 (10.00%)		
Effectivity (%)	26 (21.67%)	18 (15.00%)		
Non-effective (%)	33 (27.50%)	86 (71.67%)		
Aggravation (%)	2 (1.67%)	4 (3.33%)

(2) Effectiveness of angina pectoris symptoms

The effective rate in the experimental group was 70.83% (85 cases), whereas the effective rate in the control group was 25.00% (30 cases), with a statistically significant difference between the 2 groups (Chi-square = 50.50, *p* < 0.0001, Table 7).

**Table 7:** The effective rate of the two groups of angina pectoris symptoms.

**Project**	**Experimental group**	**Control group**	**Statistics**	***P*-value**
FAS			Chisq = 50.50	0.0000
Case (missing)	120 (0)	120 (0)		
Effectivity (%)	85 (70.83%)	30 (25.00%)		
Non-effective (%)	35 (29.17%)	90 (75.00%)

(3) Angina pectoris symptom scores at each visit point

**Figure 2:**
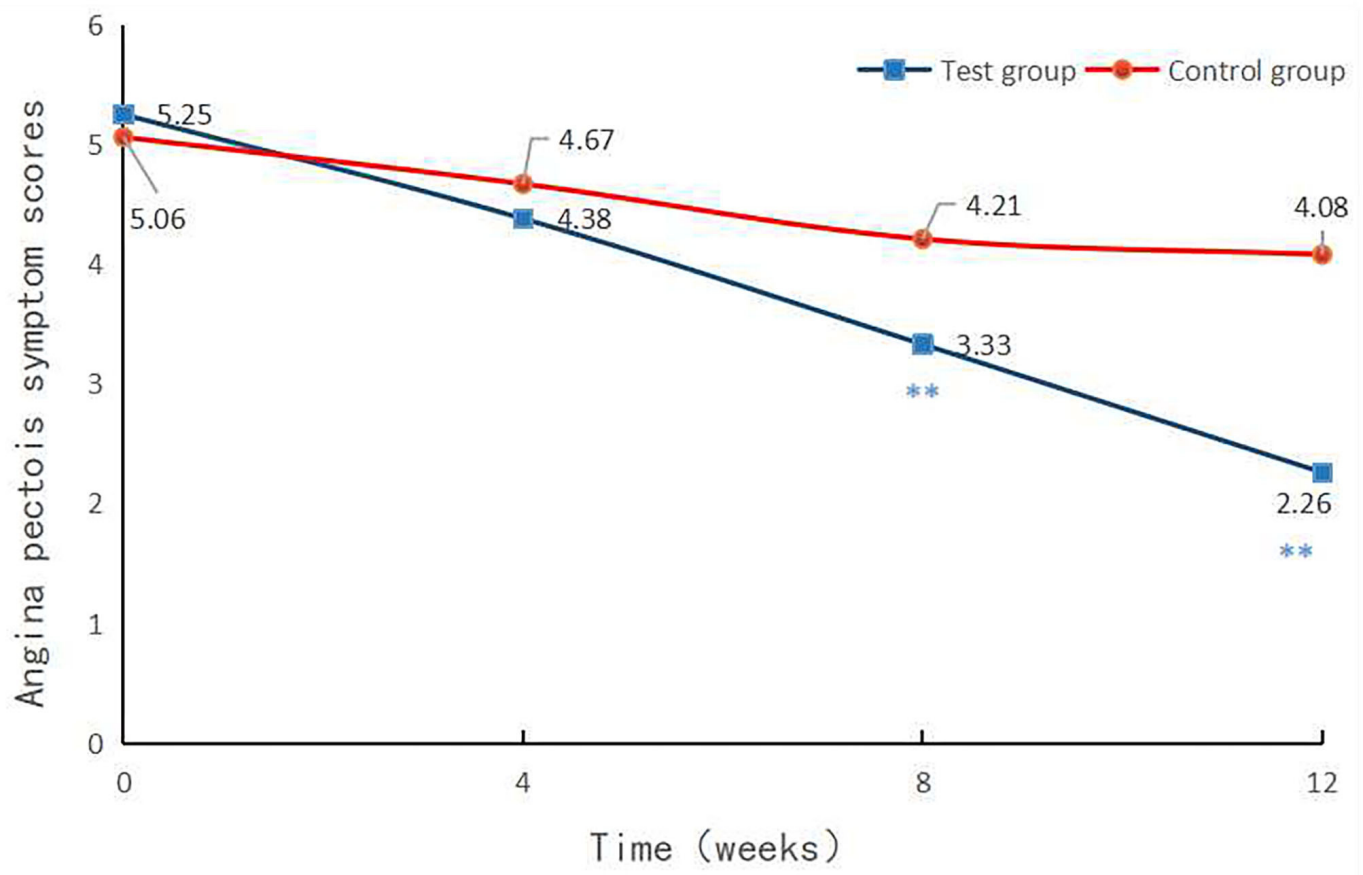
Angina pectoris symptom scores at each visit point. Angina pectoris symptom scores were compared between the two groups at baseline and in the 4th, 8th, and 12th week after treatment. *p* < 0.01.

No statistical difference was noted regarding the score of angina pectoris between the 2 groups at baseline. Following 4-week treatment, there was no statistically significant difference between the 2 groups. On the contrary, the score of angina pectoris exhibited a statistically significant difference between the 2 groups after 8- and 12-week treatment (*p* < 0.01, Figure 2).

### Results of Secondary Effectiveness Indicators

In the experimental group, the rate of 1-grade increase in severity grading reached 4.17% (5 cases), the rate of no change was 60.83% (73 cases), the rate of 1-grade reduction in severity grading was 31.67% (38 cases), and the rate of 2-grade reduction in severity grading was 3.33% (4 cases). For comparison, the control group exhibited 3.33% (4 cases) of 1-grade increase in severity grading, 75.83% (91 cases) of no change, 20.83% (25 cases) of 1-grade reduction in severity grading, and 0% (0 cases) of 2-grade reduction in severity grading. The difference between the 2 groups was statistically significant with rank-sum Z = 2.26 and *p* = 0.0240 (Table 8).

**Table 8:** Changes in the severity of angina pectoris in the two groups.

**Project**	**Experimental group**	**Control group**	**Statistics**	***P*-value**
FAS			Z = 2.26	0.0240
Case (missing)	120 (0)	120 (0)		
−1 grade (%)	5 (4.17%)	4 (3.33%)		
0 grade (%)	73 (60.83%)	91 (75.83%)		
1 grade (%)	38 (31.67%)	25 (20.83%)		
2 grade (%)	4 (3.33%)	0 (0.00%)		

#### Changes in the Number of Angina Attacks per Week

The average number of angina attacks per week was 2.28 ± 0.70 in the experimental group and 2.27 ± 0.68 in the control group, with the same median of 2. For 4-week follow-up, the mean number of angina attacks/week was 2.33 ± 0.75 in the group and 2.30 ± 0.72 in the control group. The number of weekly angina attacks elevated by 2 points in 1.72% of patients (2 cases) in the experimental group, remained unchanged in 81.90% of patients (95 cases), and decreased by 2 points in 16.38% of patients (19 cases) after 4-week treatment. As for the control group, the number of weekly angina attacks in 0.86% of patients (1 case) enhanced by 2 points, that in 91.38% of patients (106 cases) kept unchanged, and that in 7.76% of patients (9 cases) decreased by 2 points following 4-week treatment (rank-sum Z = 1.73, *p* = 0.084). As for 8-week follow-up, the average number of angina attacks was 2.26 ± 0.77 in the experimental group and 1.93 ± 1.01 in the control group. In addition, the number of weekly angina attacks in the experimental group was unchanged in 60% of patients (69 cases) and decreased by 2 points in 40% of patients (46 cases). In the control group, 79.82% of patients (91 cases) had an unchanged number of weekly angina attacks, while 20.18% of patients (23 cases) had a 2-point reduction in the number of weekly angina attacks 8 weeks after treatment (rank-sum Z = −3.26, *p* = 0.0011). After 12-week treatment, the mean number of angina attacks per week was 1.81 ± 1.01 in the experimental group and 1.60 ± 0.96 in the control group. Additionally, the weekly angina pectoris scores were unchanged in 34.23% of patients (38 cases), diminished by 2 points in 62.16% of patients (69 cases), and decreased by 4 points in 3.60% of patients (4 cases) in the experimental group. In the control group, 76.64% of patients (82 cases) displayed no change in the number of angina pectoris attacks per week, 23.36% of patients (25 cases) had a 2-point decrease in the number of angina pectoris attacks per week, and 0% of patients (0 cases) exhibited a 4-point decrease in the number of angina pectoris attacks per week 12 weeks following treatment. There was a statistically significant difference between the 2 groups (rank-sum Z = −6.36, *p* < 0.0001, Table 9).

**Table 9:** Comparison of number of angina attacks per week between the two groups (FAS).

**Index**	**Experimental group**	**Control group**	**Z**	***P*-value**
Baseline (FAS)			0.3514	0.7253
Mean ± SD	2.28 (0.70)	2.27 (0.68)		
Median	2.00 (2.00, 2.00)	2.00 (2.00, 2.00)	−0.187	0.852
Case number (missing)	120 (0)	120 (0)		
2 (%)	100 (83.33%)	102 (85.00%)		
4 (%)	20 (16.67%)	18 (15.00%)		
4-week follow-up (FAS)			−0.6927	0.4885
Mean ± SD	2.33 (0.75)	2.30 (0.72)		
Median	2.00 (2.00,2.00)	2.00 (2.00,2.00)	−0.353	0.724
Case number (missing)	120 (4)	120 (4)		
0 (%)	12 (10.34%)	7 (6.03%)		
2 (%)	89 (76.72%)	94 (81.03%)		
4 (%)	15 (12.93%)	15 (12.93%)		
Baseline vs. 4-week data (FAS)			1.728	0.0840
Case number (missing)	120 (4)	120 (4)		
−2 (%)	2 (1.72%)	1 (0.86%)		
0 (%)	95 (81.90%)	106 (91.38%)		
2 (%)	19 (16.38%)	9 (7.76%)		
Paired S-test (*p*)	93.5 (<0.0001)	22 (0.0215)		
8-week follow-up (FAS)			2.8507	0.0044
Mean ± SD	2.26 (0.77)	1.93 (1.01)		
Median	2.00 (2.00,2.00)	2.00 (2.00,2.00)	−2.683	0.007
Case number (missing)	120 (5)	120 (6)		
0 (%)	29 (25.22%)	11 (9.65%)		
2 (%)	83 (72.17%)	100 (87.72%)		
4 (%)	3 (2.61%)	3 (2.63%)		
Baseline vs. 8-week data (FAS)			−3.2608	0.0011
Case number (missing)	120 (5)	120 (6)		
0 (%)	69 (60.00%)	91 (79.82%)		
2 (%)	46 (40.00%)	23 (20.18%)		
Paired S-test (*p*)	540.5 (<0.0001)	138 (<0.0001)		
12-week follow-up (FAS)			6.3954	<0.0001
Mean ± SD	1.81 (1.01)	1.60 (0.96)		
Median	2.00 (2.00,2.00)	2.00 (2.00,2.00)	−1.942	0.052
Case number (missing)	120 (9)	120 (13)		
0 (%)	58 (52.25%)	12 (11.21%)		
2 (%)	52 (46.85%)	93 (86.92%)		
4 (%)	1 (0.90%)	2 (1.87%)		
Baseline vs. 12-week data (FAS)			−6.355	<0.0001
Case number (missing)	120 (9)	120 (13)		
0 (%)	38 (34.23%)	82 (76.64%)		
2 (%)	69 (62.16%)	25 (23.36%)		
4 (%)	4 (3.60%)	0 (0.00%)		
0 Paired S-test (p)	1,350.5 (<0.0001)	162.5 (<0.0001)		

#### Changes in Nitroglycerin Intake

Subsequent to 4- and 8-week treatment, the decrease in the nitroglycerin dosage of the patients was 0.09 ± 0.36 in the experimental group and 0.03 ± 0.25 in the control group (*p* = 0.1394). At the 12th week after treatment, the decline in the nitroglycerin dosage of the patients was 0.24 ± 0.45 in the experimental group and 0.09 ± 0.35 in the control group, with a statistically significant difference between the 2 groups (*p* = 0.0067, Table 10).

**Table 10:** Comparison of nitroglycerin intake between the two groups (FAS).

**Index**	**Experimental group**	**Control group**	***t***	***P*-value**
Baseline			*t* = 0.8694	0.3855
Case number (missing)	120 (0)	120 (0)		
Mean (±SD)	0.41 (0.51)	0.35 (0.53)		
Minimum, maximum	0, 2	0, 2		
Median (Q1, Q3)	0 (0, 1)	0 (0, 1)		
Average 95% CI	(0.32, 0.50)	(0.25, 0.45)		
4-week follow-up			*t* = 0.0000	1.0000
Case number (missing)	116 (4)	116 (4)		
Mean (±SD)	0.34 (0.51)	0.34 (0.54)		
Minimum, maximum	0, 2	0, 2		
Median (Q1, Q3)	0 (0, 1)	0 (0, 1)		
Average 95% CI	(0.24, 0.43)	(0.24, 0.44)		
Baseline vs. 4-week data			Corrected *t* = 1.4839	0.1394
Case number (missing)	116 (4)	116 (4)		
Mean (±SD)	0.09 (0.36)	0.03 (0.25)		
Minimum, maximum	−1, 1	−1, 1		
Median (Q1, Q3)	0 (0, 0)	0 (0, 0)		
Average 95% CI	(0.02, 0.15)	(−0.02, 0.07)		
Paired *t*-test (*p*)	2.56 (0.0118)	1.14 (0.2586)		
8-week follow-up			*t* = 1.4525	0.1478
Case number (missing)	115 (5)	114 (6)		
Mean (±SD)	0.23 (0.43)	0.32 (0.51)		
Minimum, maximum	0, 1	0, 2		
Median (Q1, Q3)	0 (0, 0)	0 (0, 1)		
Average 95% CI	(0.16, 0.31)	(0.23, 0.42)		
Baseline vs. 8-week data			Corrected *t* = 1.4839	0.1394
Case number (missing)	116 (4)	116 (4)		
Mean (±SD)	0.09 (0.36)	0.03 (0.25)		
Minimum, maximum	−1, 1	−1, 1		
Median (Q1, Q3)	0 (0, 0)	0 (0, 0)		
Average 95% CI	(0.02, 0.15)	(−0.02, 0.07)		
Paired *t*-test (*p*)	2.56 (0.0118)	1.14 (0.2586)		
12-week follow-up			*t* = 1.7022	0.0902
Case number (missing)	111 (9)	107 (13)		
Mean (±SD)	0.19 (0.42)	0.29 (0.46)		
Minimum, maximum	0, 2	0, 1		
Median (Q1, Q3)	0 (0, 0)	0 (0, 1)		
Average 95% CI	(0.11, 0.27)	(0.20, 0.38)		
Baseline vs. 12-week data			Corrected *t* = 2.7396	0.0067
Case number (missing)	111 (9)	107 (13)		
Mean (±SD)	0.24 (0.45)	0.09 (0.35)		
Minimum, maximum	−1, 1	−1, 1		
Median (Q1, Q3)	0 (0, 1)	0 (0, 0)		
Average 95% CI	(0.16, 0.33)	(0.03, 0.16)		
Paired *t*-test (*p*)	5.67 (<0.0001)	2.75 (0.0069)		

#### Incidence of Cardiovascular Events

The incidence of cardiovascular events in the experimental group was 0% (0 cases), and that in the control group was 0.83% (1 case), without a statistically significant difference between the 2 groups (*p* = 1.0000, Table 11).

**Table 11:** The incidence of cardiovascular events.

**Project**	**Experimental group**	**Control group**	**Statistics**	***P*-value**
FAS			Fisher	1.0000
Case (missing)	120 (0)	120 (0)		
Not occurred	120 (100%)	119 (99.17%)		
Occurred	0 (0.00%)	1 (0.83%)		

### Safety Evaluation

In the experimental group, 14 adverse events occurred in 8 cases, with an incidence rate of 6.67%. Additionally, 6 cases in the control group had 9 adverse events with an incidence rate of 5%. As per Fisher's exact probability method, the difference between the 2 groups was not statistically significant (*p* = 0.7842, Table 12).

**Table 12:** Adverse events.

	**Project**			**Experimental group**			**Comparison between two groups**
	**Times**	**Cases**	**Percentage**	**Times**	**Cases**	**Percentage**	**Fisher exact probability**
Total adverse events	14	8	6.67	9	6	5	0.7842
Major Adverse Events	0	0	0	1	1	0.83	1.0000
Serious adverse event	0	0	0	1	1	0.83	1.0000
Drug-related adverse events	6	3	2.50	6	4	3.33	1.0000
Adverse events not related to drugs	8	5	4.17	3	3	2.50	0.7219

*“Related” refers to the relationship with the study drug: “definitely related” “probably related” “may related” “suspicious”.

In 3 cases from the experimental group, 6 drug-related adverse events occurred, with an incidence rate of 2.5%, mainly including 3 cases of nausea, 1 case of vomiting, 1 case of hypoglycemia, and 1 case of stomach upset. In the control group, 4 cases had 6 drug-related adverse events, with an incidence rate of 3.33%, primarily including 3 cases of urinary tract infection, 1 case of weight loss, 1 case of hypokalemia, and 1 case of CHD. During the entire study period, there appeared no death or serious adverse event. There existed no statistically significant difference between the 2 groups before and after treatment regarding the clinical significance of the laboratory examinations (*p* < 0.05).

## Discussion

According to the “2018 China Cardiovascular Disease Report” (17), the country has 290 million patients with cardiovascular disease, with 11 million developing CHD. Cardiovascular disease accounts for a high proportion of the cost of diagnosis and treatment. In 2016, the total hospitalization cost of patients with acute myocardial infarction and acute coronary syndromes was as high as 19.085 billion RMB, ranking first among cardiovascular and cerebrovascular diseases.

TCM possesses some advantages in the prevention and treatment of CHD. With the characteristics of “simple, convenient, cheap, and effective”, TCM has demonstrated small side effects, favorable economic benefits, and definite curative effects (18). For patients with stable CHD, TCM improves the symptoms, reduces vascular restenosis and ischemia-reperfusion injury subsequent to the intervention, and represses the incidence of acute cardiovascular events (19).

The results obtained in our study found that males were prone to CSAP than females. XNNC could effectively improve angina pectoris symptoms in patients, concordant with previous studies, which affirmed the clinical efficacy of XNNC. Since the treatment period was 3 months, this study focused on the recurrence frequency and the changes in the quality of life in patients with angina pectoris to improve their quality of life. The XNNC experimental group manifested the decline in the dosage of nitroglycerin, suggesting that XNNC dilated coronary artery and ameliorated coronary artery spasm. In the 4th week of treatment, there was no statistically significant difference in the effective rate of angina pectoris between the 2 groups. However, a statistically significant difference in the 8th and 12th weeks of treatment illustrated a minimum 2-month observation period for angina pectoris symptoms. XNNC could diminish the number and duration of angina pectoris in patients. During the experiment, the incidence of cardiovascular events was 0.00% in the experimental group and 0.83% in the control group, indicating that XNNC could prevent cardiovascular events. In addition, laboratory indices in this study, such as hematuria and stool routine, adverse events, and serious adverse events, were recorded and observed before and after XNNC treatment, and the finding of no statistical difference further validated the clinical safety of XNNC.

In summary, XNNC treatment could improve the efficacy of angina pectoris, which decreased the number and duration of attacks, the dosage of nitroglycerin, and the severity of angina pain, thus protecting against cardiovascular events. Therefore, XNNC can be considered a safe and effective medicine for patients with angina pectoris.

## Data Availability Statement

The original contributions presented in the study are included in the article/supplementary materials, further inquiries can be directed to the corresponding author/s.

## Ethics Statement

This experimental study passed GCP ethics review with the ethical approval number (2017XL027-2) and clinical trial protocol registration number (NCT 03914131) (21). The patients/participants provided their written informed consent to participate in this study.

## Author Contributions

FX designed and guided this project. YL participated in research conception. PZ, LW, HW, YZ, WZ, and RG participated in clinical observation. JZ and JM collected data. JL responsible for analyzing data and drafting the article. All authors contributed to the article and approved the submitted version.

## Funding

This research was funded by the Qi-huang scholars of Chinese Medicine Inheritance and Innovation Hundred Million Talents Project (Qi-huang Project) led by FX.

## Conflict of Interest

This study received funding from Guizhou Jingcheng Pharmaceutical Co., Ltd. The funder was not involved in the study design, collection, analysis, interpretation of data, the writing of this article or the decision to submit it for publication.

## Publisher's Note

All claims expressed in this article are solely those of the authors and do not necessarily represent those of their affiliated organizations, or those of the publisher, the editors and the reviewers. Any product that may be evaluated in this article, or claim that may be made by its manufacturer, is not guaranteed or endorsed by the publisher.
